# Chronic Hyper-Hemolysis in Sickle Cell Anemia: Association of Vascular Complications and Mortality with Less Frequent Vasoocclusive Pain

**DOI:** 10.1371/journal.pone.0002095

**Published:** 2008-05-07

**Authors:** James G. Taylor, Vikki G. Nolan, Laurel Mendelsohn, Gregory J. Kato, Mark T. Gladwin, Martin H. Steinberg

**Affiliations:** 1 Pulmonary and Vascular Medicine Branch, National Heart Lung and Blood Institute, National Institutes of Health, Bethesda, Maryland, United States of America; 2 Department of Medicine, Boston University School of Medicine, Boston, Massachusetts, United States of America; 3 Critical Care Medicine Department, Clinical Center, National Institutes of Health, Bethesda, Maryland, United States of America; Leiden University Medical Center, Netherlands

## Abstract

**Background:**

Intravascular hemolysis in sickle cell anemia could contribute to complications associated with nitric oxide deficiency, advancing age, and increased mortality. We have previously reported that intense hemolysis is associated with increased risk of vascular complications in a small cohort of adults with sickle cell disease. These observations have not been validated in other populations.

**Methods:**

The distribution of serum lactic dehydrogenase (LDH) values was used as a surrogate measure of intravascular hemolysis in a contemporaneous patient group and an historical adult population from the Cooperative Study of Sickle Cell Disease (CSSCD), all with sickle cell anemia. Chronic hyper-hemolysis was defined by the top LDH quartile and was compared to the lowest LDH quartile.

**Results:**

Hyper-hemolysis subjects had higher systolic blood pressure, higher prevalence of leg ulcers (OR 3.27, 95% CI 1.92-5.53, P<0.0001), priapism (OR 2.62, 95% CI 1.13-6.90, P = 0.03) and pulmonary hypertension (OR 4.32, 95% CI 2.12-8.60, P<0.0001), while osteonecrosis (OR 0.32, 95% CI 0.19-0.54, P<0.0001) and pain (OR 0.23, 95% CI 0.09-0.55, P = 0.0004) were less prevalent. Hyper-hemolysis was influenced by fetal hemoglobin and α thalassemia, and was a risk factor for early death in the CSSCD population (Hazard Ratio = 1.97, P = 0.02).

**Conclusions:**

Steady state LDH measurements can identify a chronic hyper-hemolysis phenotype which includes less frequent vasooclusive pain and earlier mortality. Clinicians should consider sickle cell specific therapies for these patients, as is done for those with more frequent acute pain. The findings also suggest that an important class of disease modifiers in sickle cell anemia affect the rate of hemolysis.

## Introduction

Hemolysis is a pathologic mechanism leading to cardiovascular, pulmonary, gastrointestinal, and renal manifestations in diverse human diseases [1]. Such complications have been attributed to vascular nitric oxide (NO) depletion via direct scavenging reactions with cell free plasma hemoglobin and impaired NO generation due to enzymatic consumption of arginine by red cell arginase I [1]–[3].

These sequelae are putative risk factors for morbidity and mortality in aging populations with congenital hemolytic anemias like sickle cell anemia (homozygous *HBB* val6). In adult sickle cell disease, pulmonary hypertension has a high prevalence and mortality [4], [5]. A potential mechanism for its development is pulmonary vasoconstriction and cellular proliferation in response to decades of chronic hemolysis associated with reduced vascular NO [1], [2], [4], [6], [7]. Priapism and leg ulcers are also associated with markers of hemolysis leading to speculation that these, and perhaps other disease manifestations, like stroke, could also be a consequence of hemolysis-induced NO deficiency [4], [7]–[9]. Prior work from our group reported that hemolysis, as estimated by plasma LDH level and its red cell derived isoforms, might identify a cardiovascular sub-phenotype among adults with heterogeneous forms of sickle cell disease [7].

While prior epidemiologic studies have focused on individual disease manifestations, we hypothesized that the degree of hemolysis is a key determinant influencing a phenomic spectrum of complications that reflect the severity of sickle vasculopathy [10]–[13]. Our objective was to refine our previously reported subphenotype [7], now limited to patients with sickle cell anemia, and to validate the findings in a larger independent population. Using two separate adult populations, we examined the extremes of LDH distributions in sickle cell anemia to determine if intravascular hemolysis represents a unifying phenotype. Our results suggest that chronic excess, or hyper-hemolysis, is associated with a vascular phenotype in adults that is distinguishable from sickle vasoocclusive complications like acute painful events and osteonecrosis, and is associated with premature mortality.

## Methods

### Study Populations

NIH sickle cell patients evaluated between February, 2001 and June, 2007 were screened for pulmonary hypertension in an NHLBI IRB approved protocol (ClinicalTrials.gov Identifier: NCT00011648) which included written informed consent [4], [6], [7]. Subject recruitment continued both from local clinics and through media advertisements [4]. Two hundred sixty three sickle cell anemia subjects identified by sequencing the *HBB* locus, with or without coincident α thalassemia, were included [14]. Data from the Cooperative Study of Sickle Cell Disease (CSSCD; ClinicalTrials.gov Identifier: NCT00005277) was used to independently validate findings. This natural history study followed more than 4000 subjects between 1978 and 1988 [15]. Hydroxyurea treatment was not available, pulmonary hypertension screening by transthoracic echocardiography was not done routinely and measures of intravascular hemolysis and NO physiology were not part of the original protocol. CSSCD patients with sickle cell anemia, with or without coincident α thalassemia, were included in the present study.

### Selection of Study Patients (Figure 1)

In NIH patients, LDH values were measured during steady state at initial evaluation with no acute crises or transfusion in the 2 weeks prior to evaluation. No subject had evidence of acute hyper-hemolysis due to crisis, infection or hemolytic transfusion reaction. In CSSCD subjects, we used the median LDH value from steady state measurements taken over 6 years of observation. Patients with ALT values >80 IU were also excluded to avoid confounding by hepatopathy with hepatic LDH elevation. To approximate the demographics of subjects at NIH, and to specifically test the effect of hemolytic rate on cardiovascular complications which evolve with increasing age, CSSCD subjects selected were aged greater than 30 years [4], [14]. This age filter generated an older CSSCD study population that was comparable to the NIH; one third of both study populations were comprised of subjects aged more than 45 years (Figure S1). Because the CSSCD recruited patients of all ages, there was an extreme bias towards younger patients without this selection criteria (only 623 or 23% were aged more than 30 years versus 150 or 57% at the NIH, P<0.0001). Within each population, LDH was divided into quartiles based on the LDH distribution; the lowest and highest quartiles (representing values greater than the 75^th^ percentile or less than the 25^th^ percentile) were compared in subsequent analyses.

**Figure 1:**
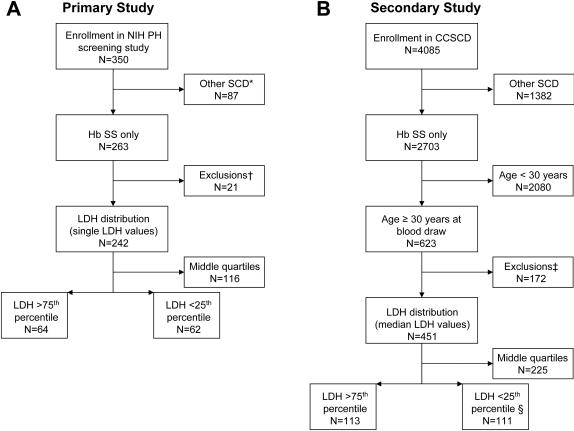
Selection of adult sickle cell anemia subjects for LDH analysis. Panel A: Selection process in the NIH study that identified subjects with the highest and lowest LDH values. * Includes both sickle cell disease cases and 10 subjects for whom no DNA sample was collected. † Of the 21 subjects excluded from analysis, 14 had an excessive hemolysis index for LDH, 2 had no labs drawn, 4 had ALT values greater than 80 IU/L, and 1 had aplastic anemia. Panel B: Selection in the validation study from the CSSCD. ‡ 172 had ALT values greater than 80 IU/L. § Two were excluded with unexpectedly low LDH values.

### Clinical Definitions

At NIH, acute chest syndrome (ACS) was defined as a patient reported history of one or more episodes of “chest syndrome” or “pneumonia” requiring hospitalization. Stroke was defined by patient reported cerebrovascular disease with some documented by MRI. Osteonecrosis was defined by a history of osteonecrosis of the femoral head or joint replacement. Pain was quantified by average emergency room visits per year for evaluation of severe episodes of acute sickle cell related pain or by categorical comparisons (no annual severe pain episodes versus any severe episodes). In NIH subjects, pulmonary hypertension was defined by tricuspid regurgitant jet velocity (TRV) or N-terminal pro brain naturetic peptide (NTproBNP) levels as described previously, and severe pulmonary hypertension represented a TRV greater than or equal to 3.0 m/s [4], [6]. CSSCD definitions for ACS, stroke, osteonecrosis, and pain are reported elsewhere, and used confirmed clinical criteria [10], [13], [16], [17]. NTproBNP levels were measured in a subset of the CSSCD high and low LDH groups by immunoassay (Elecsys Analyzer; Roche Diagnostics, Maneheim, Germany) from stored, frozen plasma samples. Glomerular filtration rate was calculated using the Cockroft-Gault formula [18].

### Statistical Analysis

Analyses were performed using Instat (Graph Pad Software, San Diego, CA), Prism (Graph Pad Software), or SAS version 8.2 (SAS Institute, Cary, NC). Comparison of categorical data was by Fisher exact test (2×2 table with 1 degree of freedom) including odds ratios (ORs) and 95% confidence intervals (CIs). Combined analyses were stratified and are reported as Mantel-Haenszel (MH) weighted Odds Ratios and P values derived from MH summary chi-squares. Continuous data were log transformed where appropriate and analyzed by paired t test, Alternate Welch’s t test, or Mann-Whitney test. In this study, P values ≤0.05 were considered significant.

## Results

### LDH as a Continuous Trait and Chronic Hyper-Hemolysis in the NIH Population

**Figure 2:**
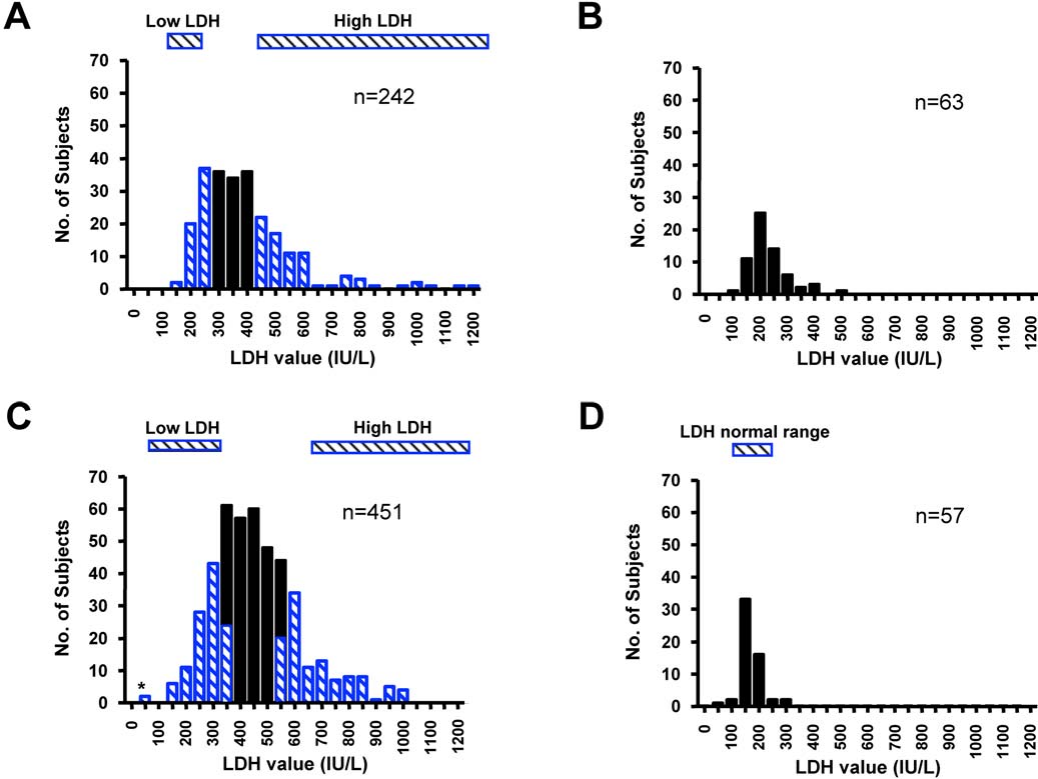
LDH distributions in sickle cell anemia. Panel A: LDH distribution at NIH. Blue bars indicate either the Low LDH study group defined by LDH below 278.0 IU/L or the high LDH group (LDH above 451.0 IU/L). Panel B: LDH distribution among NIH subjects with Hemoglobin SC disease and sickle β^+^ thalassemia. Panel C: Distribution of median LDH values from up to three LDH measurements in the CSSCD. The blue bars show the Low LDH (at or below the LDH 25th percentile of 340.5 IU/L) and the High LDH (at or above the 75th percentile of 546.0 IU/L) study groups, respectively. * Two were excluded from the Low LDH group for unexpectedly low LDH values. Panel D: LDH distribution among healthy controls.

LDH values were examined as a quantitative trait (Figure 2A). For comparison, LDH values from 63 NIH subjects with sequence verified hemoglobin SC disease or sickle β^+^ thalassemia (Figure 2B) and 57 healthy African American controls (Figure 2D) demonstrate considerable overlap with values for the NIH sickle cell anemia low LDH group (Figure 2A) [19]. Sixty four sickle cell anemia subjects had LDH values greater than the 75^th^ percentile and 62 had values less than the 25^th^ percentile (Figure 2A, Table 1). Inclusion in the highest LDH quartile defined chronic hyper-hemolysis.

**Table 1:** Laboratory Characterization of the High and Low Hemolysis Phenotypes of Sickle Cell Anemia.

Parameter	NIH			CSSCD		
	High LDH	Low LDH	P value*	High LDH	Low LDH	P value*
	Mean (SD) or No. (%)	Mean (SD) or No. (%)		Mean (SD) or No. (%)	Mean (SD) or No. (%)	
	N = 64	N = 62		N = 113	N = 111	
LDH, U/L	610.6 (178.4)	234.5 (30.5)	-	680.8 (126.8)	276.0 (49.3)	-
Total bilirubin, mg/dL	3.8 (1.8)	2.4 (1.4)	<0.0001	3.9 (2.3)	2.5 (1.5)	<0.0001
Direct bilirubin, mg/dL	0.7 (0.5)	0.4 (0.3)	<0.0001	ND	ND	ND
ALT, U/L	28.5 (11.1)	26.0 (15.8)	0.02	31.2 (19.4)	26.1 (16.3)	0.08
AST, U/L	56.5 (18.1)	33.0 (16.8)	<0.0001	58.9 (25.5)	34.5 (16.4)	<0.0001
Hemoglobin, g/dL	7.9 (1.5)	9.4 (1.6)	<0.0001†	8.4 (1.5)	8.7 (1.4)	0.01
Hematocrit, %	22.4 (5.0)	27.7 (4.8)	<0.0001†	23.0 (3.6)	26.5 (4.2)	<0.0001
MCV, fL	92.9 (10.7)	95.8 (12.6)	0.16†	94.2 (7.8)	93.1 (8.7)	0.43
Absolute reticulocytes, 10^9^/L§	275.2 (130.2)	230.6 (123.9)	0.06	ND	ND	ND
Reticulocytes, %	N/A	N/A	N/A	11.3 (5.1)	11.0 (4.9)	0.70
Fetal hemoglobin, %	6.7 (5.2)	10.7 (7.5)	0.005	5.4 (5.3)	6.8 (5.3)	0.01
Fetal hemoglobin, g/dL	0.6 (0.5)	1.0 (0.8)	0.0006	0.5 (0.5)	0.6 (0.5)	0.007
Hemoglobin A, %	12.5 (20.5)	10.1 (18.8)	0.51†	ND	ND	ND
>5% Hemoglobin A, No. (%)	23 (36%)	18 (29%)	0.41	ND	ND	ND
Arginine, µmol/L	39.2 (14.5)	45.8 (16.2)	0.01	ND	ND	ND
Arginine:Ornithine ratio	0.67 (0.33)	0.82 (0.36)	0.02‡	ND	ND	ND
Arginase 1 activity, µmol/mL/hr	3.66 (2.76)	1.39 (0.70)	0.001‡	ND	ND	ND
Plasma hemoglobin, µmol/L	21.2 (18.3)	12.1 (15.4)	0.0003	ND	ND	ND
Plasma VCAM-1, ng/mL	1449.9 (773.1)	966.7 (642.6)	<0.0001	ND	ND	ND
Ferritin, µg/L	672 (847)	1017 (1352)	0.25	ND	ND	ND
WBC, 10^9^/L	10.4 (3.1)	10.0 (3.4)	0.32†	11.5 (2.7)	11.4 (2.9)	0.80
C-reactive protein, mg/dL	0.63 (0.84)	0.83 (1.24)	0.62	ND	ND	ND

Abbreviations: NIH, National Institutes of Health; CSSCD, Cooperative Study of Sickle Cell Disease; High LDH, patients with LDH values >75^th^ percentile; Low LDH, patients with LDH values <25^th^ percentile; ND, not determined.

* Mann-Whitney nonparametric test unless otherwise indicated.

† Unpaired t test.

‡ Unpaired t test with Welch correction.

§ Absolute reticulocytes were not directly measured in the CSSCD.

Laboratory characteristics were then compared between LDH groups (Table 1). Subjects with chronic hyper-hemolysis had significantly higher levels of total bilirubin, AST, arginase 1 activity, plasma hemoglobin and plasma vascular cell adhesion molecule-1. This group also had lower hemoglobin concentrations, serum arginine, arginine to ornithine ratios, and fetal hemoglobin (HbF) levels. There were no significant differences in mean HbA levels due to recent transfusion, or in the proportion of subjects who had received episodic red cell transfusion therapy with greater than 5% HbA (Table 1). To determine if the difference in HbF could be attributed to treatment with hydroxyurea, as suggested by the difference in hydroxyurea prescription rates between the LDH groups (Table 2, P = 0.03), all subjects receiving hydroxyurea or with more than 5% HbA (from transfusion) were censored from a second HbF analysis, leaving 83 eligible subjects, 21 each in the high and low LDH quartiles (Table S1). This eliminated any suggestion of an MCV difference (Table S2, P = 0.98), and the mean HbF was again lower in the high LDH group (HbF 0.4±0.4 gm/dL) than in the low LDH group (HbF 0.8±0.5 gm/dL) (Table S2, P = 0.06). This smaller population limited statistical power, but did not change the magnitude of differences between the groups for other labs or clinical complications (Table S2 and S3).

**Table 2:** Clinical Associations with the Hyper-Hemolysis Phenotype in Sickle Cell Anemia.

Clinical Variable	NIH					CSSCD				
	High LDH		Low LDH		P value	High LDH		Low LDH		P value
	N = 64	No. (%) or Mean (SD)	N = 62	No. (%) or Mean (SD)		N = 113	No. (%) or Mean (SD)	N = 111	No. (%) or Mean (SD)	
Male	64	31 (48%)	62	25 (40%)	0.38	113	62 (55%)	111	29 (26%)	<0.0001
Age, yrs.	64	36.3 (11.9)	62	34.7 (12.3)	0.36*	113	41.1 (8.8)	111	39.0 (7.6)	0.06
SBP, mmHg	53	121.5 (17.5)	54	115.2 (17.8)	0.05*	113	113.5 (12.0)	111	110.8 (12.4)	0.02
DBP, mmHg	53	65.8 (11.3)	54	65.4 (9.7)	0.99*	113	68.7 (77.8)	111	68.5 (8.7)	0.67
SpO_2_, %	46	94.6 (3.7)	44	97.9 (2.3)	<0.0001*	ND	ND	ND	ND	ND
Haptoglobin†	54	0 (0%)	45	5 (10%)	0.02	ND	ND	ND	ND	ND
α thalassemia	52	17 (33%)	49	22 (45%)	0.20‡	113	15 (13%)	111	41 (37%)	<0.0001
Osteonecrosis	57	6 (11%)	56	15 (27%)	0.07‡	113	30 (27%)	111	59 (53%)	<0.0001
ACS	61	47 (77%)	56	48 (86%)	0.41‡	113	80 (71%)	111	80 (72%)	0.83
Leg ulcers	58	17 (29%)	55	8 (15%)	0.05‡	113	56 (52%)	111	25 (23%)	<0.0001
Priapism (male)	27	15 (56%)	23	6 (26%)	0.04‡	62	20 (32%)	30	6 (20%)	0.32
Stroke	59	12 (20%)	57	7 (12%)	0.39‡	113	10 (9%)	111	5 (5%)	0.29
GFR, mL/min.	58	127.0 (59.7)	52	142.4 (63.0)	0.19	83	105.9 (49.1)	88	104.1 (35.2)	0.92
ER visits/yr.§	46	2.5 (4.7)	46	6.9 (14.3)	0.004*	113	1.1 (2.3)	111	1.4 (2.6)	0.10
≥1 Pain events/yr.	46	24 (52%)	46	39 (85%)	0.005‡	113	102 (90%)	111	108 (97%)	0.05
Hydroxyurea	59	23 (39%)	58	36 (62%)	0.03	ND	ND	ND	ND	ND
>10 Transfusions	53	20 (38%)	50	23 (46%)	0.40	ND	ND	ND	ND	ND
PH	64	41 (58%)	62	27 (38%)	0.01‡	ND	ND	ND	ND	ND
Severe PH	64	23 (36%)	62	6 (10%)	0.002‡	ND	ND	ND	ND	ND
Elevated BNP	59	31 (53%)	60	11 (18%)	0.0001‡	41	21 (51%)	40	10 (25%)	0.02

Abbreviations: SBP, systolic blood pressure; DBP, diastolic blood pressure; α thalassemia refers to co-existing heterozygosity or homozygosity for α^3.7^ (genotypes αα/-α^3.7^ and -α^3.7^/-α^3.7^ combined); ACS, acute chest syndrome as a prevalence; GFR, glomerular filtration rate in mL/min.; PH, pulmonary hypertension defined by triscuspid regurgitant jet velocity (TRJV) ≥2.5 m/s; severe PH, severe pulmonary hypertension defined by TRJV ≥3.0 m/s; Elevated BNP, N-terminal pro brain naturetic peptide ≥160 pg/mL and ND, not determined. The total number of subjects in each study group is listed at the top of the table along with the actual number of subjects with available data for each parameter.

* Mann Whitney nonparametric test.

† Reported as the number of subjects with detectable haptoglobin levels (greater than 6 mg/dL).

‡ Adjusted for hydroxyurea exposure.

§ Emergency room visits only for the evaluation of severe episodes of acute sickle cell related pain.

### Clinical Manifestations Associated with Chronic Hyper-Hemolysis in the NIH Population

Hyper-hemolysis clinical characteristics were compared with the low LDH group (Table 2). Chronic hyper-hemolysis subjects had higher systolic blood pressures (P = 0.05) and lower pulse oximetry measurements (P<0.0001). Haptoglobin was undetectable in hyper-hemolysis patients compared to 5 subjects with measurable levels among low LDH subjects (mean 70.6 mg/dL, n = 5, OR 0.07, 95% CI 0.04-1.26, P = 0.02, Table 2). These were 5 of only 6 detectable haptoglobin levels within the NIH population where this was measured (n = 198), including one 65 year old female without detectable HbA or prior treatment with hydroxyurea. Chronic hyper-hemolysis subjects had more priapism (P = 0.04), pulmonary hypertension (P = 0.01), severe pulmonary hypertension (P = 0.002), and elevated NTproBNP levels (P = 0.0001). Chronic hyper-hemolysis subjects also had fewer annual emergency room visits for acute painful episodes (P = 0.004). This is consistent with a significantly lower prevalence for hydroxyurea prescriptions (OR 0.40, 95% CI 0.19-0.85, P = 0.03) (Table 2).

### Stability of Steady State Hemolytic Rate and Validation in the CSSCD Population

**Figure 3:**
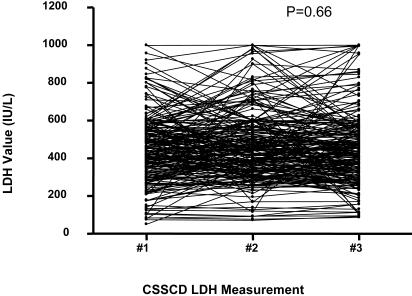
Stability of steady state LDH values from the CSSCD. The stability of steady state hemolytic rate is demonstrated by analysis of 3 serial LDH measurements in 225 CSSCD subjects (repeated measures of ANOVA, P = 0.66).

To validate these associations, an independent analysis was performed in the CSSCD. The distribution of median LDH values for 451 subjects identified 113 in the top quartile and 111 in the bottom quartile (Figure 2C). Two subjects in the bottom quartile were excluded due to outlying low LDH values. In addition, a single LDH determination reflected a relatively constant rate of hemolysis in an individual at steady state, based upon analysis of 225 (49%) of the CSSCD subjects with 3 serial LDH measurements (Figure 3; repeated measures of ANOVA, P = 0.66). Subjects with the highest LDH values again had significantly more corroborative evidence of hemolysis and lower HbF levels (Table 1).

When the clinical characteristics of the high and low LDH groups were compared, a phenotypic spectrum of complications similar to those identified in the NIH population was observed (Table 2). Among the chronic hyper-hemolysis group, there was a significantly higher systolic blood pressure (P = 0.02), more prevalent leg ulcers (P<0.0001), a lower prevalence of coincident α thalassemia (genotypes αα/-α^3.7^ and -α^3.7^/-α^3.7^; P<0.0001), less severe acute pain (P = 0.05) and a lower prevalence of osteonecrosis (P<0.0001). There was also a male predominance in the hyper-hemolysis group (P<0.0001). Because there were significant differences in the sex distribution and HbF levels between LDH comparison groups in the CSSCD (Tables 1 and 2), Odds Ratios were adjusted for sex and/or HbF, where appropriate. Adjustments changed neither the significance nor magnitude of the associations (data not shown).

### Clinical Complications in the NIH, CSSCD and Combined Populations

**Figure 4:**
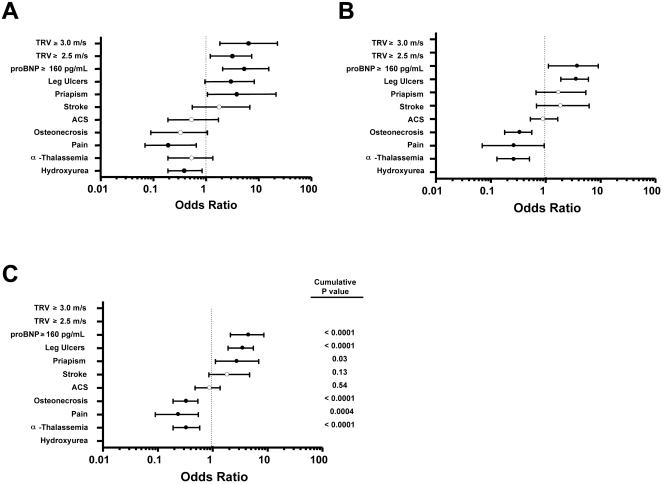
Clinical manifestations associated with hyper-hemolysis in sickle cell anemia. Panel A: Odds ratios and confidence intervals for associations with hemolysis in the NIH study. • = P value ≤0.05; ○ = P value not significant. Panel B: Associations in the CSSCD population. Panel C: Summary odds ratios for associations between hyper-hemolysis and 8 clinical endpoints in a combined analysis of the NIH and CSSCD populations.

Odds ratios for selected clinical complications comparing LDH groups for the NIH, CSSCD, and combined populations are presented in Figure 4 and Table S4. Combined results show that leg ulcers (OR 3.27, 95% CI 1.92-5.53, P<0.0001), priapism (OR 2.62, 95% CI 1.13-6.90, P = 0.03) and pulmonary hypertension defined by NTproBNP (OR 4.32, 95% CI 2.12-8.60, P<0.0001) were significantly more prevalent with chronic hyper-hemolysis, while osteonecrosis (OR 0.32, 95% CI 0.19-0.54, P<0.0001) and severe pain episodes (OR 0.23, 95% CI 0.09-0.55, P = 0.0004) were less prevalent (Figure 4C). There was no significant difference in acute chest syndrome (OR 0.83, 95% CI 0.49-1.41, P = 0.54) or stroke (OR 1.95, 95% CI 0.85-4.72, P = 0.13). Hyper-hemolysis was also associated with a significantly lower prevalence of α thalassemia (OR 0.33, 95% CI 0.19-0.58, P<0.0001).

### Survival with Chronic Hyper-Hemolysis

**Figure 5:**
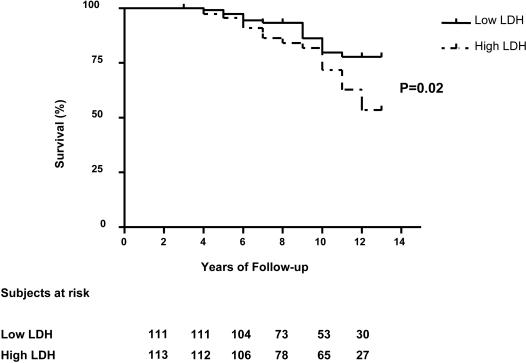
Hyper-hemolysis is associated with early mortality in the CSSCD. Kaplan Meier survival curve for the CSSCD according to LDH group for 224 subjects. Early mortality was associated with the high LDH group by logrank test (Hazard ratio 1.97, 95% confidence interval 1.14–3.41, P = 0.02).

High LDH values were associated with higher mortality in the entire NIH cohort [7]. We therefore examined the extremes of hemolytic rate from the CSSCD population for effects of LDH distributions on mortality. Chronic hyper-hemolysis was associated with earlier mortality in the CSSCD population (Figure 5, Hazard ratio 1.97, 95% CI 1.14-3.41, log-rank test P = 0.02), where the mean follow-up was 10.2 years.

## Discussion

Identification of genetic variants underlying human disease has become a priority [20]. A major limitation for genetic studies is that phenotypes defined by a medical diagnosis are often imprecise. This recognition led to a proposed Human Phenome Project, where phenomics would examine both inter-individual variability in newly recognized phenotypes and correlations between different subphenotypes within a single disease [21]. We propose that chronic intravascular hyper-hemolysis represents a novel sickle cell anemia subphenotype. Its identification might be helpful for locating disease modifying factors and developing new therapeutic strategies.

Sickle cell anemia is a prototypical hemolytic disorder. Nevertheless, the degree of hemolysis varies among patients and its intensity is modulated by HbF expression and co-incident α thalassemia. It is also likely to be influenced by other genes important in sickle cell pathophysiology. Based on an analysis of steady state LDH distributions in nearly 700 adults with sickle cell anemia, chronic hyper-hemolysis represents a phenotype in independent populations who were recruited originally for different purposes during different therapeutic eras. Individuals with hyper-hemolysis had less frequent vasoocclusive pain and osteonecrosis, increased prevalence of leg ulcers and priapism, higher systolic blood pressure and more pulmonary hypertension. Earlier mortality also characterized hyper-hemolysis. Unexpectedly, hyperhemolysis did not result in significantly more reticulocytosis in either population, as was also observed in our prior analysis of pulmonary hypertension in sickle cell anemia [14]. Reticulocytosis is a physiologic response to overall levels of hemolysis, including both extravascular and intravascular red cell destruction. Thus, we speculate that the similar reticulocytes levels might be explained by our inability to quantify differences in the degree of ineffective erythropoiesis, levels of extravascular hemolysis, or their collective effects on reticulocytosis between LDH groups. As expected, the hyper-hemolysis phenotype was also associated with lower HbF levels and a lower prevalence of α thalassemia. These findings provide additional insight into some of the factors that affect severity and overall survival, and could impact indications for treatment.

Alpha thalassemia is a known genetic determinant that lessens anemia and hemolysis (Table 2) [22], [23]. It is associated with less priapism [8], fewer leg ulcers [11], [24], and more osteonecrosis [16], [24], [25], while there have been conflicting results with respect to vasoocclusive pain [10], [13], [24], [26]. Protective associations with priapism and leg ulcers are consistent with effects on bioavailable NO [1], [2]. Alpha thalassemia was significantly less prevalent among those with hyper-hemolysis further implicating effects on NO as a potential mechanism underlying these associations. Similarly, high levels of HbF are associated with less frequent pain, acute chest syndrome, leg ulcers, and reduced mortality [9]–[13]. This is consistent with HbF’s ability to inhibit HbS polymerization, thereby decreasing either vasoocclusion or hemolysis [27]. Surprisingly, associations have not been observed between HbF and priapism or pulmonary hypertension [4], [8]. In this study, higher HbF and fewer vascular complications were found in the low LDH groups. Perhaps HbF has a sufficiently small protective effect against individual complications such that an association is only detectable through secondary analysis of closely related traits like hemolysis. Identifying such effects is further complicated by the variable distribution of HbF among F-cells which was not assayed in either study population. Putative loci controlling cellular HbF distribution have been localized to chromosomes 2p, 6q, 8q and Xp22, and in particular, the absence of X inactivation at Xp22 results in higher F-cells in females [28]–[31]. Additional studies of X-linked genes for HbF or glucose-6- phosphate dehydrogenase might explain the male predominance in the CSSCD chronic hyper-hemolysis group.

Previous studies have suggested the possibility of a chronic hyper-hemolysis phenotype [13], [32]–[36]. Comparison of sickle cell anemia in Greece and Jamaica suggested that leg ulcers and anemia were much more common in Jamaica [35]. Ballas proposed that similar observations could represent different sickle cell phenotypes, one defined by leg ulcers, and the other by frequent pain. Infrequent pain was also associated with decreased red cell deformability and more dense erythrocytes [33]. Finally, Bayesian modeling was used to dichotomize the Jamaican cohort study into either pain crisis or leg ulcer phenotypes, where leg ulcers were associated with anemia [32]. In fact, the paradoxical relationship between prominent anemia and fewer pain crises is a consistent finding in multiple studies in agreement with our conclusion that chronic hyper-hemolysis may be characterized by fewer pain crises [13], [34], [36].

Several limitations are inherent to our studies. First, the CSSCD, whose patients were enrolled more than 25 years ago, had only a limited number of older adults. Additional retrospective analysis of infrequent adult complications like stroke is limited by this demographic, although the combined analysis could suggest a trend towards an association between stroke and hemolysis when interpreted in the context of other works [19], [37], [38]. A high proportion of CSSCD subjects were excluded due to an elevated ALT (Figure 1), suggesting that subclinical liver disease (e.g., viral hepatitis) was more common than it is presently with universal hepatitis B vaccination and screening of blood for hepatitis C [39]. Also, hydroxyurea therapy was not available, while its potential beneficial effects might be evident in the low LDH group at NIH. Early mortality with hyper-hemolysis was evident with long term follow-up in the larger CCSCD population, and presumably this was due to previously unrecognized pulmonary hypertension as suggested by the higher prevalence of elevated NTproBNP levels. The survival difference was only apparent after 6 years, suggesting that longer follow-up at NIH will be necessary to confirm this finding [7]. We have also shown that markers of hemolysis are a disease component forecasting earlier death using network modeling [40].

Chronic hyper-hemolysis can identify individuals at risk for premature mortality who may be treated with hydroxyurea less often than patients with frequent pain, its major clinical indication. Despite prior findings that hydroxyurea has no beneficial effect on pulmonary hypertension, its use has been advocated on the basis of improved erythrocyte survival and experiential treatment in hemoglobinopathy clinics [4], [5], [41]. Thus, prospective long term clinical trials evaluating the effects of hydroxyurea, transfusion therapy and other innovative treatment modalities focused on preserving NO bioavailability and decreasing hemolysis, cell free hemoglobin and vascular complications are warranted [41]–[43].

These data support an emerging model for understanding the protean manifestations of sickle cell anemia that could be influenced by either hemolysis-driven vasculopathy or blood viscosity/vasoocclusion [44]. Future genetic studies of this unifying phenotype might provide novel mechanistic insights into pathogenesis and treatment. Clinically, this study further supports the use of steady state LDH measurements to identify sickle cell anemia patients who are at increased risk for earlier mortality and who might otherwise have treatment options overlooked because of infrequent vasoocclusive pain.

## Supporting Information

Figure S1 Figure S1 with supplementary data.
(0.10 MB TIF)

Table S1 LDH Quartile Analysis for Untransfused Sickle Cell Anemia Patients Not Taking Hydroxyurea.
(0.03 MB DOC)

Table S2 Laboratories in Untransfused NIH Sickle Cell Anemia Subjects Not Taking Hydroxyurea.
(0.05 MB DOC)

Table S3 Clinical Associations in Untransfused NIH Sickle Cell Anemia Subjects Not Taking Hydroxyurea.
(0.05 MB DOC)

Table S4 Clinical Associations with Hyper-Hemolysis in Sickle Cell Anemia.
(0.06 MB DOC)
