# Supplementary material for: Chronic Hyper-Hemolysis in Sickle Cell Anemia: Association of Vascular Complications and Mortality with Less Frequent Vasoocclusive Pain
Source: PLoS One. 2008 May 7;3(5):e2095. doi: 10.1371/journal.pone.0002095 (PMC2330070; doi:10.1371/journal.pone.0002095)
Supplement: Table S1 — LDH Quartile Analysis for Untransfused Sickle Cell Anemia Patients Not Taking Hydroxyurea. (0.03 MB DOC) [file pone.0002095.s002.doc]

**Table S1**. LDH Quartile Analysis for Untransfused Sickle Cell Anemia Patients Not Taking Hydroxyurea.

| **LDH Category** | **Hb SS only***  **LDH (n=83)** |
| --- | --- |
| Minimum  25^th^%ile  Median  75^th^%ile  Maximum | 201.0  305.5  390.0  481.0  1177.0 |

* Patients taking either hydroxyurea or with detectable Hb A were excluded from the analysis. From a total of 83 HbSS patients, 21 were identified in the high and low LDH quartiles, above the 75^th^ percentile and below the 25^th^ percentile, respectively.
