# Supplementary material for: Chronic Hyper-Hemolysis in Sickle Cell Anemia: Association of Vascular Complications and Mortality with Less Frequent Vasoocclusive Pain
Source: PLoS One. 2008 May 7;3(5):e2095. doi: 10.1371/journal.pone.0002095 (PMC2330070; doi:10.1371/journal.pone.0002095)
Supplement: Table S2 — Laboratories in Untransfused NIH Sickle Cell Anemia Subjects Not Taking Hydroxyurea. (0.05 MB DOC) [file pone.0002095.s003.doc]

**Table S2. Laboratories in Untransfused NIH Sickle Cell Anemia Subjects Not Taking Hydroxyurea**.

| **Parameter** | **NIH cohort** | | |
| --- | --- | --- | --- |
|  | **High LDH**  **Mean (SD)**  **(n=21)** | **Low LDH**  **Mean (SD)**  **(n=21)** | **P value*** |
| LDH, U/L | 642.5 (182.8) | 250.3 (27.0) | - |
| Total bilirubin, mg/dL | 4.7 (2.0) | 2.8 (1.4) | 0.001† |
| Direct bilirubin, mg/dL | 0.7 (0.5) | 0.4 (0.2) | 0.03 |
| ALT, U/L | 29.0 (11.0) | 25.5 (14.5) | 0.17† |
| AST, U/L | 60.2 (18.7) | 37.8 (18.8) | <0.0001 |
| Hemoglobin, g/dL | 7.4 (1.1) | 9.2 (1.4) | <0.0001† |
| Hematocrit, % | 21.1 (3.5) | 26.9 (4.2) | <0.0001† |
| MCV, fL | 88.7 (10.3) | 88.6 (13.4) | 0.98† |
| Absolute reticulocytes, 10^9^/L | 351.4 (89.2) | 282.9 (133.9) | 0.06† |
| Fetal hemoglobin, % | 5.7 (4.5) | 7.9 (4.9) | 0.14 |
| Fetal hemoglobin, g/dL | 0.4 (0.4) | 0.8 (0.5) | 0.06 |
| Arginine, μmol/L | 39.6 (13.7) | 49.5 (13.6) | 0.03† |
| Arginine:Ornithine ratio | 0.67 (0.26) | 0.90 (0.35) | 0.02† |
| Arginase 1 activity, μmol/mL/hr | 3.70 (3.20) | 1.57 (0.38) | 0.07‡ |
| Plasma hemoglobin, μmol/L | 24.6 (18.4) | 14.2 (20.4) | 0.002 |
| Plasma VCAM-1, ng/mL | 1195.0 (228.2) | 907.0 (444.4) | 0.006 |
| Ferritin, μg/L | 196 (204) | 572 (830) | 0.06 |
| WBC, 10^9^/L | 10.6 (2.8) | 11.4 (2.9) | 0.38† |
| C-reactive protein, mg/L | 5.6 (8.7) | 6.3 (7.7) | 0.47 |

* Mann-Whitney nonparametric test unless otherwise specified.

† Unpaired t test.

‡ Alternate Welch’s t test.
