# Supplementary material for: Chronic Hyper-Hemolysis in Sickle Cell Anemia: Association of Vascular Complications and Mortality with Less Frequent Vasoocclusive Pain
Source: PLoS One. 2008 May 7;3(5):e2095. doi: 10.1371/journal.pone.0002095 (PMC2330070; doi:10.1371/journal.pone.0002095)
Supplement: Table S3 — Clinical Associations in Untransfused NIH Sickle Cell Anemia Subjects Not Taking Hydroxyurea. (0.05 MB DOC) [file pone.0002095.s004.doc]

**Table S3. Clinical Associations in Untransfused NIH Sickle Cell Anemia Subjects Not Taking Hydroxyurea.**

| **Clinical Variable** | **NIH cohort** | | | |
| --- | --- | --- | --- | --- |
|  | **High LDH**  **No. (%) or Mean (SD)**  **(n=21)** | **Low LDH**  **No. (%) or Mean (SD)**  **(n=21)** | **OR (95% CI)*** | **P value†** |
| Male (%) | 11 (52%) | 7 (33%) | - | 0.35 |
| Age, years | 36.6 (11.3) | 35.6 (13.7) | - | 0.64 |
| SBP, mmHg | 118.2 (15.7) | 116.5 (10.1) | - | 0.71 |
| DBP, mmHg | 60.7 (5.4) | 63.4 (8.0) | - | 0.25 |
| SpO_2_, % | 92.0 (3.7) | 97.7 (1.9) | - | <0.0001 |
| Haptoglobin† | 0 (0%) | 1 (5%) | 0.35 (0.01-9.19) | 1.00 |
| α thalassemia | 3 (18%) | 5 (36%) | 0.39 (0.07-2.03) | 0.41 |
| Osteonecrosis | 4 (19%) | 7 (37%) | 0.40 (0.10-1.69) | 0.29 |
| ACS | 17 (81%) | 16 (80%) | 1.06 (0.23-4.98) | 1.00 |
| Leg ulcers | 6 (30%) | 4 (21%) | 1.61 (0.37-6.92) | 0.72 |
| Priapism (male) | 4 (44%) | 1 (17%) | 4.00 (0.32-49.62) | 0.58 |
| Stroke | 3 (14%) | 2 (10%) | 1.50 (0.22-10.08) | 1.00 |
| GFR, mL/min. | 119.9 (45.3) | 133.63 (82.2) | - | 0.59 |
| ER visits/yr.‡ | 2.8 (6.0) | 3.3 (6.3) | - | 0.84 |
| ≥ 1 Pain events/yr. | 8 (50%) | 12 (75%) | 0.33 (0.07-1.49) | 0.27 |
| PH | 11 (52%) | 9 (43%) | 1.50 (0.43-4.95) | 0.76 |
| Severe PH | 6 (29%) | 2 (10%) | 3.80 (0.67-21.61) | 0.24 |
| Elevated BNP | 10 (48%) | 4 (20%) | 3.64 (0.90-14.61) | 0.10 |

* Odds Ratio (95% Confidence Interval).

† Mann-Whitney nonparametric test.

‡ Emergency room visits only for the evaluation of severe episodes of acute sickle cell related pain.
