# Supplementary material for: Chronic Hyper-Hemolysis in Sickle Cell Anemia: Association of Vascular Complications and Mortality with Less Frequent Vasoocclusive Pain
Source: PLoS One. 2008 May 7;3(5):e2095. doi: 10.1371/journal.pone.0002095 (PMC2330070; doi:10.1371/journal.pone.0002095)
Supplement: Table S4 — Clinical Associations with Hyper-Hemolysis in Sickle Cell Anemia. (0.06 MB DOC) [file pone.0002095.s005.doc]

**Table S4. Clinical Associations with Hyper-Hemolysis in Sickle Cell Anemia.**

| **Clinical Variable** | **NIH cohort**  **Without Adjustment** | **NIH cohort***  **Adjusted** | **CSSCD cohort** | **Combined†**  **Without Adjustment** | **Combined***  **Adjusted** |
| --- | --- | --- | --- | --- | --- |
| Severe pulmonary hypertension  Odds Ratio (95% CI)  P value | 5.24 (1.96-14.02)  0.0006 | 6.30 (1.85-22.87)  0.002 | ND | ND | ND |
| Pulmonary hypertension  Odds Ratio (95% CI)  P value | 2.31 (1.13-4.73)  0.03 | 2.92 (1.22-7.47)  0.01 | ND | ND | ND |
| NTproBNP > 160 pg/mL  Odds Ratio (95% CI)  P value | 4.93 (2.15-11.31)  0.0001 | 5.55 (2.08-15.72)  0.0003 | 3.15 (1.12-9.09)  0.02 | 4.06 (2.09-7.92)  <0.0001 | 4.32 (2.12-8.60)  <0.0001 |
| Leg ulcers  Odds Ratio (95% CI)  P value | 2.44 (0.95-6.23)  0.07 | 2.97(0.96-8.34)  0.05 | 3.38 (1.90-6.03)  <0.0001 | 3.08 (1.84-5.23)  <0.0001 | 3.27 (1.92-5.53)  <0.0001 |
| Priapism  Odds Ratio (95% CI)  P value | 3.54 (1.07-11.77)  0.05 | 4.19 (1.07-21.51)  0.04 | 1.91 (0.67-5.40)  0.32 | 2.47 (1.07-6.11)  0.04 | 2.62 (1.13-6.90)  0.03 |
| Stroke  Odds Ratio (95% CI)  P value | 1.82 (0.66-5.03)  0.32 | 1.86 (0.55-6.84)  0.39 | 2.06 (0.68-6.23)  0.29 | 1.93 (0.87-4.45)  0.12 | 1.95 (0.85-4.72)  0.13 |
| Acute chest syndrome  Odds Ratio (95% CI)  P value | 0.56 (0.21-1.46)  0.25 | 0.59 (0.19-1.73)  0.41 | 0.93 (0.53-1.67)  0.83 | 0.81 (0.48-1.37)  0.49 | 0.83 (0.49-1.41)  0.54 |
| Osteonecrosis  Odds Ratio (95% CI)  P value | 0.32 (0.11-0.90)  0.03 | 0.33 (0.09-1.07)  0.07 | 0.32 (0.18-0.56)  <0.0001 | 0.32 (0.19-0.54)  <0.0001 | 0.32 (0.19-0.54)  <0.0001 |
| Pain  Odds Ratio (95% CI)  P value | 0.20 (0.07-0.53)  0.002 | 0.21 (0.07-0.66)  0.005 | 0.26 (0.07-0.95)  0.05 | 0.22 (0.09-0.50)  0.0001 | 0.23 (0.09-0.55)  0.0004 |
| α –thalassemia  Odds Ratio (95% CI)  P value | 0.60 (0.27-1.34)  0.23 | 0.51 (0.19-1.35)  0.20 | 0.26 (0.13-0.51)  <0.0001 | 0.36 (0.21-0.61)  <0.0001 | 0.33 (0.19-0.58)  <0.0001 |
| Hydroxyurea treatment  Odds Ratio (95% CI)  P value | 0.40 (0.19-0.85)  0.03 | ND | ND | ND | ND |

Abbreviations and definitions: All comparisons are for High LDH vs. Low LDH; Pulmonary hypertension is defined as a tricuspid regurgitant jet velocity of ≥ 2.5 m/s; Severe pulmonary hypertension is defined as a tricuspid regurgitant jet velocity of ≥ 3.0 m/s; ACS, acute chest syndrome; Pain is defined by the presence of one or more severe acute vasoocclusive crisis episodes requiring treatment in an emergency room or hospital; ND = not determined.

* Values adjusted for hydroxyurea exposure with Mantel-Haenszel weighted Odds Ratios and Mantel-Haenszel summary chi-squares.

† Mantel-Haenszel weighted Odds Ratios with exact 95% confidence intervals and P values determined from Mantel-Haenszel summary chi-squares to combine the study populations.
